# The composition of the gut microbiome differs among community dwelling older people with good and poor appetite

**DOI:** 10.1002/jcsm.12683

**Published:** 2021-02-13

**Authors:** Natalie J. Cox, Ruth C.E. Bowyer, Mary Ni Lochlainn, Philippa M. Wells, Helen C. Roberts, Claire J. Steves

**Affiliations:** ^1^ Academic Geriatric Medicine, Faculty of Medicine University of Southampton Tremona Road Southampton UK; ^2^ NIHR Southampton Biomedical Research Centre University of Southampton and University Hospital Southampton NHS Foundation Trust Southampton UK; ^3^ Department of Twins Research and Genetic Epidemiology, Kings College London St Thomas' Hospital London UK; ^4^ NIHR Applied Research Collaboration (ARC) Wessex Southampton UK; ^5^ Department of Ageing and Health Guy's and St Thomas' NHS Foundation Trust London UK

**Keywords:** Gut microbiome, Appetite, Sarcopenia, Nutrition, Older people

## Abstract

**Background:**

Anorexia of ageing is common and important in the development of sarcopenia in older individuals. Links have been proposed between the gut microbiota and sarcopenia. Disordered gut function is also recognized in anorexia of ageing, but how this may relate to resident gut microbiota is unexplored. Understanding this relationship may provide a basis for novel interventions for anorexia of ageing and sarcopenia. This study explores compositional differences of the gut microbiota between community dwelling healthy older adults with good or poor appetite, and associated differences in sarcopenia.

**Methods:**

We assessed appetite by the Simplified Nutritional Appetite Questionnaire (SNAQ) in members of the TwinsUK cohort aged ≥65 years. Using a pool of 776 individuals with existing microbiome data estimated from 16S rRNA sequencing data, we identified 102 cases (SNAQ score < 14) (95% female, mean age 68 years) matched to controls (SNAQ > 14) on body mass index, gender, age, diet, calorie consumption, frailty, antibiotic use, socio‐economic status, and technical variables to minimize confounding microbiota associations. Species abundance and diversity, compositional differences, and paired differences in taxa abundance were compared between cases and controls. Additionally, we compared case and controls for sarcopenia as measured by muscle mass (appendicular lean mass/height^2^) and strength (chair stand time in seconds).

**Results:**

Cases with poor appetite had reduced species richness and diversity of their gut microbiome (adjusted OBSERVED: beta = −0.2, *P* < 0.001; adjusted SHANNON: beta = −0.17, *P* = 0.0135), significant compositional differences (adjusted non‐parametric multivariate analysis of variance, *P* = 0.0095), and significant differences in taxa abundance including reduction of genus *Lachnospira* (logFC = −1.015, *q* = 0.023). In all‐female subgroup analysis, cases with poor appetite demonstrated reduction in muscle strength (11.03 s vs. 9.26 s, *P* = 0.02).

**Conclusions:**

This study is the first to observe differences in the composition of gut microbiota between healthy community dwelling older individuals with good and poor appetite. We found female individuals with reduced muscle strength had poor appetite compared with those with normal strength. These associations require further examination to understand causality and mechanisms of interaction, to inform potential strategies targeting the gut microbiota as a novel intervention for anorexia of ageing and sarcopenia.

## Introduction

Appetite is the collection of sensations that drive consumption of food.^1^ Loss of appetite, or anorexia, is a common occurrence in older people, estimated to affect 27% of those aged over 65 years in the community,^2^ rising to 41% in acute care.^3^ This has important consequences, most notably as a major component in the development of sarcopenia,^4^ a progressive skeletal muscle disorder involving the accelerated loss of muscle mass and function.^5^

Loss of appetite in older individuals can often be attributed to medical conditions, such as chronic obstructive pulmonary disease, heart or renal failure, and medication including opiates and certain antimicrobials among others.^1^ However, it is also due to multiple underlying determinants as part of the ageing process in the anorexia of ageing.^1^, ^6^ This includes well‐recognized alterations in mechanical and neuroendocrine functioning of the gut.^4^, ^7^ An integral component of gut neuroendocrine activity is the interaction with resident gut microbiota (bacteria, archaea, viruses, and eukaryotic microbes). For example, microbial metabolites, including short‐chain fatty acids (SCFAs), branched chain amino acids, and peptidoglycans, are critical to enterocyte function, including interacting with enteroendocrine cells in the gut wall to affect signalling.^8^, ^9^, ^10^ The composition of the gut microbiome is known to change with age and health status.^11^ Notably, alterations in its composition have been associated with frailty, anabolic resistance, and skeletal muscle function.^12^, ^13^ For example, a randomized controlled trial of a prebiotic, which modulates the gut microbiota, in older adults, reported improvements in two of Fried's frailty criteria^14^ (exhaustion and grip strength) and a reduction in a frailty index for those given the prebiotic vs. those given the placebo.^15^, ^16^ Research has found that frailty is negatively associated with the diversity of the gut microbiota.^12^, ^17^ This has led researchers to propose that composition and diversity of the gut microbiome may be considered a biomarker and possible influencer of healthy ageing.^18^

Despite a shared association with major health burdens in the older population, including sarcopenia, the relationship between the gut microbiome and appetite loss in older people is currently unexplored. The primary objective of this study was therefore to explore if there was a difference in the composition of the gut microbiota between community dwelling people aged 65 years and older reporting good or poor appetite. We used a case–control matching strategy with known microbiota correlates to reduce variance due to confounding and technical factors. Secondarily, in a subgroup of cases and controls, we explored differences in muscle mass and muscle strength as markers of sarcopenia between the two groups.

## Methods

### Study sample

Participants were members of the TwinsUK, the UK's largest research cohort of adult twins.^19^ The cohort was started in 1992 and now incorporates approximately 14 000 community dwelling twins, who are predominantly female (82%).

### Data collection

#### Appetite

Appetite was assessed using the Simplified Nutritional Appetite Questionnaire (SNAQ),^20^ distributed in an online format. This is a simple questionnaire comprising 4 Likert scale items covering appetite and intake, which has been validated for use in community dwelling older people.^20^ The online questionnaire was open to responses from 5 March to 30 April 2019 and sent to 2183 members of the TwinsUK cohort aged 65 years and older.

#### Microbiota collection and analysis

Microbiome data for members of the cohort had been collected and sequenced prior to the online questionnaire on a rolling basis between 2010 and 2015 (with most samples collected between 2013 and 2015). Microbiome composition was assessed using 16S rRNA gene metabarcoding. The 16S rRNA gene is a highly conserved region of the bacterial genome, which contains several hypervariable regions that allow for broad differentiation between microbial species; we sequenced the V4 region. Processing and sequencing of the samples used in this study has been described previously.^21^ Briefly, participants collected samples at home and stored them in sealed ice packs until either providing them at a clinical visit or posting them with 24 h of delivery. Samples were homogenized, aliquoted, and frozen at −80°C upon receipt. An aliquot of ~100 mg was shipped to Cornell University for DNA extraction, PCR amplification, and sequencing using the pipeline described in Goodrich et al 2011. Amplicons were sequenced using 250 bp paired‐end sequences on the Illumina MiSeq platform.

Amplicon sequences were processed into amplicon sequence variance (ASVs) using the DADA2 pipeline,^22^ following demultiplexing in Qiime.^23^ Forward and reverse read files were generated, quality trimming and error estimation for each sample, and then the ASV algorithm applied, after which the forward and reverse ASVs were joined. This process was run separately for each sequencing run. Next, data sets were merged, and sequences collapsed, chimeras removed, with taxonomic information assigned using the SILVA 1.3.2 database and phylogenetic tree generated using the phangorn package.^24^, ^25^ ASVs can be considered as a microbial ‘unit’ as best identified by 16S rRNA sequence.

#### Markers of sarcopenia

Muscle mass was assessed by dual‐energy X‐ray absorptiometry on members of the cohort between 2006 and 2018 (majority of assessments between 2013 and 2018) and calculated as appendicular lean mass/height^2^. Muscle strength was assessed using chair stand time, with data collected for members of the cohort between 2013 and 2018 (with an even spread of collection over the years). European Working Group on Sarcopenia in Older People 2 (EWGSOP2) guidance^5^ was used to define low muscle mass (<0.6 kg/m^2^ for female participants) and low muscle strength (chair stand time > 15 s for five rises).

### Case–control matching and analysis of participants with appetite and microbiome data

Frailty of responders to the online questionnaire were compared with non‐responders to assess the potential for healthy selection bias using a questionnaire‐derived frailty index.^26^ Frailty was root normalized and compared between responders and non‐responders using Welch two sample *t*‐test. Cases and controls were matched from within the pool of questionnaire responders with existing microbiota data (*n* = 776). For those with multiple microbiota samples, the sample nearest to SNAQ response was used. The use of case–control approach to analysis limits bias from unbalanced response data and reduced variance from other variables associated with the microbiota that could be acting as confounders. Using a threshold previously established by Wilson *et al*,^20^ we considered anyone with a SNAQ score below 14 (indicating poor appetite) as cases and anyone scoring above 14 as controls. We discounted those scoring 14 to aid disparity between those reporting good and poor appetite.

Controls were matched on variables known to be associated with microbiota. A Euclidean distance matrix was calculated on body mass index (BMI—m^2^/kg) at microbiome sample, sex (coded as binary), age at microbiome sample, diet (as measured by the Healthy Eating Index 2010 and calculated from Food Frequency Questionnaire—Bowyer *et al* ^27^), calorie consumption (kcal/day, estimated from Food Frequency Questionnaire), frailty (measured by Rockwood's Frailty Index^26^), antibiotics use in the month prior to sample (coded as binary), decile representing the Index of Multiple Deprivation downloaded from National websites, the difference in time between microbiota sample and SNAQ, and sequencing depth (i.e. the number of viable sequences identified within each sample). Measurements for BMI were taken at the clinical visit (where microbiome sample was provided). Health deficit and dietary data were from questionnaire data, collected as part of rolling data collections; the measurement temporally closest was used. Missing data had previously been imputed using the MissForest package in R.^28^ Controls were matched to cases as the sample with the shortest Euclidean distance to the case. Where two cases were matched to the same control, one was randomly reassigned to the control with the next shortest distance, until all cases were matched to unique controls. Paired Wilcoxon‐rank sum tests were used to assess success of case–control matching for all variables.

Microbiota variance in association with SNAQ was assessed in three ways. First, measures of species diversity (Shannon Diversity, Observed species, and Inverse Simpsons index) were used as the response variables in linear models, with the inclusion of sequencing depth (a technical measure relating to the number of viable sequences in a sample) and family structure (a measure to control for the relatedness of participants) as covariates. Models were repeated with additional adjustment for Healthy Eating Index 2010, age, (root normalized) frailty, time between samples, gender, and use of antibiotics in month prior to sample. All variables were scaled and centred prior to the model; null models were repeated without the SNAQ variable of interest; analysis of variance of the results are reported. Standardized coefficients were calculated using the lm.beta package.^29^

Secondly, the intraindividual dissimilarity of microbiota was calculated (i.e. how different one sample is from all of the others). ASV tables were trimmed to those prevalent in at least 5% of the population subset and transformed using the negative binomial.^30^ The weighted UniFrac distance was calculated on the resulting count table. Difference in microbiota composition for case and controls was compared using non‐parametric multivariate analysis of variance (NPMANOVA) (ran with 2000 permutations) and homogeneity of variance tests using the Vegan package.^31^, ^32^ NPMANOVA was repeated, adjusted for covariates. The average within‐group distances for case and control was calculated, as was the distance between case and corresponding control, and compared using two‐sample Wilcoxon tests.

Finally, paired tests of differences in taxa abundances were used to assess difference in members of the microbial community between cases and controls. EdgeR^33^ was used to calculate the difference at ASV level, and where count tables had been grouped at genus and phylum levels.

### Comparison of markers of sarcopenia between cases and controls

A subgroup analysis was performed on cases and controls with data on markers of sarcopenia (muscle mass and muscle strength), to assess if there were any differences between those with good and poor appetite.

## Results

### Population characteristics

The online questionnaire was distributed to 2183 individuals aged over 65, of which 1494 returned responses (68.4%). Of those sent the questionnaire, 1923 had available frailty index data and were used to compare health‐deficit between responders and non‐responders, with suggestion of some healthy responder bias to the questionnaire, as the frailty of responders (*μ* = 0.44, *σ* = 0.1, *n* = 1419) was lower than those who did not respond (*μ* = 0.46, *σ* = 0.1, *n* = 504; two sample *t*‐test *t*(845) = −3.98, *P* < 0.001).

Of the 1494 responders to the online SNAQ, 757 had existing microbiota data (of which 211 had multiple samples). This resulted in 102 cases with a SNAQ score < 14 and available microbiota data, which were matched to controls with a SNAQ score > 14 and available microbiota data. Distributions and case–control matching of covariates are in Supporting Information, *Figure* S1; population characteristics are in *Table* 1. Paired Wilcoxon‐rank sum tests were not significant between cases and controls for any of the considered covariates; this suggests efficacy of our case–control matching strategy. In the majority of case–control pairs, an unrelated individual was the nearest match with only four cases being matched with their cotwin on key variables. Overall, the sample was predominantly female in gender, with a mean age of 67 years, mean BMI of 25 and a frailty index of 0.2.

**Table 1:** Population characteristics of cases vs. controls

Variable	Case	Control
Total number	*n* = 102	*n* = 102
SNAQ (*μ*, *σ*)	12.078 (1.31)	16.186 (0.982)
Gender (%F)	95.09	95.09
Age (*μ*, *σ*)	67.97 (5.67)	67.64 (5.43)
BMI (kg/m^2^) (*μ*, *σ*)	25.55 (5.1)	25.76 (4.1)
HEI (*μ*, *σ*)^a^	59.98 (11.44)	59.96 (10.37)
Calorie Consumption (kcal/day)	1828.098 (608.44)	1862.19 (476.79)
FI (*μ*, *σ*)^a^	0.209 (0.1)	0.2 (0.09)
IMD decile (*μ*)^b^	6.83 (2.49)	7.11 (2.34)
Antibiotics use^a^ (%No)	100	100
Difference in sample time (microbiome and SNAQ) (years) (*μ*, *σ*)	5.6 (1.25)	5.52 (1.25)
Sequencing depth (*μ*, *σ*)	85 531 (41 851)	83 437 (41 570)

BMI, body mass index; FI, Rockwood frailty index; HEI, Healthy Eating Index 2010; IMD, index of multiple deprivation; SNAQ, Simplified Nutritional Appetite Questionnaire.

^a^ Includes imputed variables.

^b^ Ordinal variable treated as continuous for descriptives.

### Case–control analysis of appetite and microbiome

**Figure 1:**
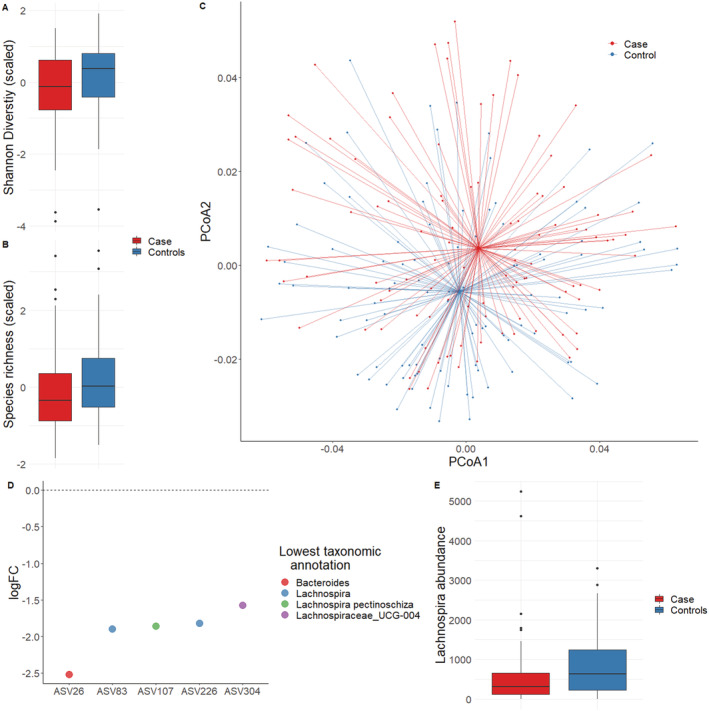
Microbiota differences observed between cases (Simplified Nutritional Appetite Questionnaire < 14) and controls (Simplified Nutritional Appetite Questionnaire > 14). (A) Shannon diversity (scaled) and (B) microbiota richness (scaled) between cases and controls. (C) First two PCoAs of weighted UniFrac distances, with case and control group centroids. (D) False discovery rate‐significant results of paired‐tests of differences in ASV abundance between cases and controls and (E) difference in the *Lachnospira* genus, observed as false discovery rate‐significant in paired‐tests of differences in genus abundance.

Of the three species diversity indexes considered (Shannon diversity, Observed species, and Inverse Simpson diversity), none were significant in simple paired tests, but observed species and Shannon diversity were significantly different between cases and controls in linear regression models adjusted for library size and family structure (SHANNON: beta = 0.17, *P* = 0.016, OBSERVED: beta = −0.2, *P* = 0.001); the association remained in models adjusted for covariates (SHANNON: beta = − 0.17, *P* = 0.0135, OBSERVED: beta = −0.2, *P* < 0.001) (*Figure* 1A–1B). This suggests species richness and ASV abundances are reduced between cases and controls.

There were significant differences in microbiota composition between cases and controls in models including library size (NPMANOVA, *P* = 0.013, permutations = 2000, *Figure* 1C); the association remained when variance was constrained for considered covariates (Methods) (NPMANOVA, *P* = 0.0095, permutations = 2000). Post‐hoc tests for homogeneity of variance were not significant suggesting differences between case and control were not confounded by differences in group dispersion. Paired distances (weighted UniFrac) of case and their corresponding control were not significantly different than within‐case or within‐control distances (pairwise Wilcoxon‐rank sum test); this suggests within‐group dissimilarity does not account for the differences we observe (*Figure* S2).

In paired test of differences in microbiota abundances, of the 771 ASVs considered, five were significant following false discovery rate (FDR) adjustment; all ASVs were reduced in cases. Of the five significant (*q* < 0.05), only one had taxonomic information at species level, an ASV assigned as *Lachnospira pectinoschiza*. The remaining had genus level annotation: two as *Lachnospira*, one *Bacteroides*, and one as Lachnospiraceae *UCG‐004* (*Table* 2, *Figure* 1D, ASV sequences and results in *Data* S3A).

**Table 2:** Paired tests of difference in ASV abundances between case and control (logFC represents change in case compared with control)

ASV id	Family	Genus	Species	LogFC	FDR‐*P* value
ASV26	Bacteroidaceae	*Bacteroides*	Unassigned	−2.518	<0.001
ASV107	Lachnospiraceae	*Lachnospira*	*pectinoschiza*	−1.855	0.0015
ASV226	Lachnospiraceae	*Lachnospira*	Unassigned	−1.821	0.0016
ASV83	Lachnospiraceae	*Lachnospira*	Unassigned	−1.575	0.0032
ASV304	Lachnospiraceae	*Lachnospiraceae_UCG‐004*	Unassigned	−1.575	0.009

FDR, false discovery rate; ASV, amplicon sequence variance.

ASV sequences are in *Data* S3A.

Of the 156 genus considered, and reflecting the ASV level analysis¸ *Lachnospira* was FDR‐significantly reduced in cases (logFC = −1.015, *q* = 0.023, *Figure* 1E, *Data* S3B) and of the 11 phyla considered Tenericutes was FDR‐significantly reduced in cases (logFC = −1.576, *q* = 0.0379.

### Case–control comparison of markers of sarcopenia

Data on muscle strength were available for 114 individuals (56%), all of whom were female. One hundred and five (51%) individuals had available data on muscle mass, again all were female.

Cases had a significantly higher mean chair stand time compared with controls, indicating reduced muscle strength (11.03 vs. 9.26 s, *P* = 0.02, *Table* 3). There was no significant difference between mean muscle mass of cases and controls. Ninety‐nine (87%) individuals had reduced muscle strength, and 61 (58%) had reduced muscle mass as defined by EWGSOP2.^5^ Of individuals with reduced muscle strength, a higher number were cases (SNAQ < 14) [*n* = 57 (57.6%), *P* = 0.02]. There was no significant difference between number of cases and controls with reduced muscle mass (33 vs. 28, *P* = 0.6).

**Table 3:** Case–control comparison of sarcopenia markers

Marker of sarcopenia	Cases (*n* = 53)	Controls (*n* = 61)	*P*
Chair stand time (s) (*μ*, *σ*)	11.03 (4.98)	9.26 (2.51)	0.02
Muscle mass (appendicular lean mass/height^2^) (*μ*, *σ*)	5.82 (0.69)	6.02 (0.80)	0.6

## Discussion

In the present study, we explored differences in the composition of the gut microbiome between cases with poor appetite and controls with a good appetite, in a community dwelling sample of mainly female members of the TwinsUK cohort aged 65 and older. Cases were matched to controls on variables known to associate with and likely influence microbiota composition including diet, frailty, socio‐economic status, and antibiotic use; this approach reduces microbiota variance attributable to other factors and balances the study design. Our findings demonstrate that there are marked differences in microbiota composition between cases with poor appetite and controls with good appetite. To our knowledge, this is the first description of an association between poor appetite and variability in the composition of the gut microbiota in community dwelling older individuals. Secondly, in this study, we sought to determine if there was a difference in markers of sarcopenia between those with good or poor appetite. We demonstrated that those with poor appetite had reduced muscle strength measured by increased chair stand time, when compared with controls with good appetite.

Anorexia drives sarcopenia via the development of undernutrition and loss of muscle mass.^4^, ^34^, ^35^ However, sarcopenia independent of overall weight loss has been observed in community dwelling older people with anorexia,^36^ and anorexia predicts muscle strength independent of muscle mass in older people post‐hospital discharge.^37^ Our analysis is consistent with these findings as we identified that individuals with reduced muscle strength had poor appetite compared with those with normal strength. This difference was significant despite the small sample size. There was no difference in muscle mass between cases and controls in our analysis. Of note, the most recent EWGSOP2 guidelines recommend using muscle strength as more meaningful marker of sarcopenia than mass.^5^ Muscle mass varies depending on the method used to measure it, overidentifies those who are slim as having sarcopenia, and can underidentify sarcopenia in those who have large mass, which includes fat, known as sarcopenic obesity.^5^

The gut microbiota plays an integral role in absorption and metabolism of components in an individual's diet, affecting their biochemical profile,^38^ and so impacting on both quantitative and qualitative nutritional health. We observe a clear difference in microbiota composition between cases and controls; including the suggestion of a reduction in *Lachnospira* in cases. *Lachnospira* have been associated with exercise training‐induced improvements in muscle strength, and some species of the genus are a notable butyrate producing bacteria (a SCFA). Higher levels of butyrate have been suggested as the mechanism linking dietary fibre intake with higher percentage of whole body lean mass and physical functioning in older adult men.^39^, ^40^ Importantly, SCFAs produced by Lachnospiraceae have also been associated with reduction in inflammatory bowel disease via interactions inducing regulatory T cells with anti‐inflammatory functions.^41^ *Lachnospira* abundance is associated with vegetable‐rich/high‐fibre diets (particularly vegetarian and vegan diets).^42^, ^43^, ^44^ Alterations in dietary pattern are seen in older people with poor appetite, including reduction in fresh vegetables.^45^, ^46^ Appetite change with dietary alteration may therefore predispose to qualitative malnutrition, with reductions in specific macronutrients or micronutrients affecting muscle health and contributing to the onset of sarcopenia.^36^ Our findings may add to this theory with recognition that those with poor appetite have an altered microbiota composition and reduction in *Lachnospira*, which is associated with vegetable rich diets. However, in our study, cases and controls were matched on dietary similarity via a healthy eating index, which includes a vegetable intake domain. This highlights the complexity of host–microbiota interaction, with possible alternate mechanisms impacting upon *Lachnospira* abundance beyond dietary intake alone.^18^ Conversely, it may also reflect a limitation of using self‐report mechanisms for capturing dietary data.^47^

We also observed microbiota species richness and diversity were reduced in cases compared with controls. Although differences in species (or alpha) diversity should be interpreted with caution,^48^ in general lower relative species, diversity is thought to be broadly associated with ill‐health. The ELDERMET study reported significantly lower diversity of gut microbiota composition between those in a care‐home setting vs. matched community dwellers.^17^ Furthermore, age‐associated alterations in intestinal microbiota composition are implicated in age‐related decline of immune system function and low grade chronic inflammation (immunosenecence and inflammaging), along with anabolic resistance to dietary protein in the elderly.^13^, ^49^

**Figure 2:**
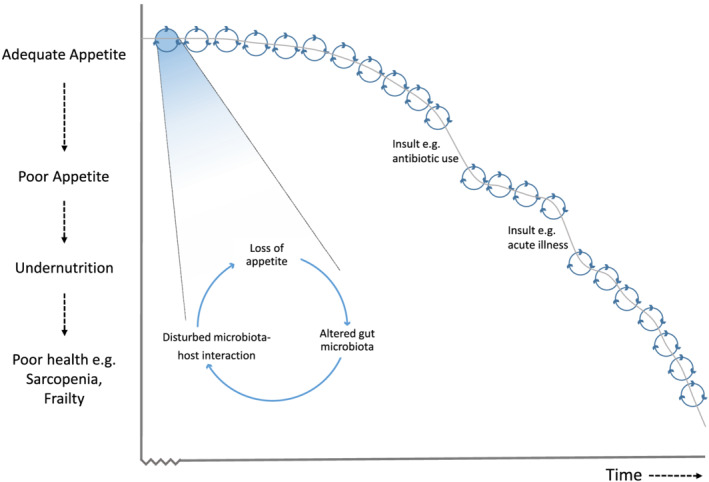
Theoretical cyclical relationship between appetite change and the gut microbiome in the trajectory over time to worse health outcomes.

While shifts in microbiota composition could simply still be reflective of the overall reduced health, and/or reduced fibre intake in cases compared with controls, the fact that we found striking difference in microbiota with appetite after carefully matching on health status and diet would certainly suggest further investigation of a potentially causal relationship. The observed changes in the gut microbiota in our analysis were from samples pre‐dating the appetite assessment by up to 8 years. This raises the possibility that disturbed host–microbiota functioning is implicated in appetite loss. Indeed, the modification of gut hormone secretion by bacteria, including glucagon‐like peptide‐1 (GLP‐1), peptide YY, leptin and ghrelin, suggests the mechanism could be via disruptive bacterial signals in these key neuroendocrine interactions.^50^ Particularly, alterations in circulating levels of GLP‐1, peptide YY, and Ghrelin have been observed in older individuals.^4^, ^7^, ^51^ On the other hand, changes to appetite in older individuals may be influencing dietary consumption, further impacting on the observed differences in overall community composition and microbial abundances in the microbiome. Certainly, gut microbiota diversity has previously been associated with both healthy^27^ and a varied diet.^52^ Therefore, there may be a potentially cyclical relationship between microbiome alterations and the development of ill‐health over time, with loss of appetite leading to altered gut microbiota, leading to disturbed microbiota–host interaction, leading to loss of appetite and so on (*Figure* 2). Stepwise acceleration in the trajectory to worse health outcomes may occur given insults such as acute illness (*Figure* 2)—certainly, poor appetite during hospital stay is associated with increased mortality in the 6 months post‐hospital discharge.^3^

## Study limitations

This study was in a predominantly female cohort, so further analysis is needed representative of both genders. As appetite data were not collected at the same time as faecal sample, it is unclear whether appetite was also poor at the time of faecal collection, or whether gut dysbiosis may be a pre‐cursor to subsequent poor appetite. However, daily calorific intake (measured concurrently to microbiome sample) was matched between cases and controls and there was no difference between the two groups (*Figure* S1). The data on muscle strength and mass were collected at a prior time‐point, so it is again unclear whether appetite was also poor at this point, or prior to it, as previous studies suggest.

Another limitation to this study was the potential risk of healthy responder bias to the online appetite questionnaire. However, this was mitigated by the case–control analysis, which included matching for frailty. The use of self‐reported questionnaires to derive measures of frailty and diet is a limitation, as well as the lack of detailed information on medications other than antibiotic use, which may affect the gut microbiota (such as proton pump inhibitor or laxative use). While self‐report approaches enable the study of larger populations, future targeted studies should consider measures of objective frailty and monitor daily dietary consumption. The microbiota analysis utilized 16S rRNA metabarcoding rather than full metagenomic analysis, which would give a more comprehensive view of microbiota profile.

## Conclusion and future directions

To our knowledge, this is the first demonstration of a difference in composition of the gut microbiome between older individuals with good and poor appetite. Individuals with a poor appetite had reduced species richness and diversity, in particular reduction of *Lachnospira*. Furthermore, demonstrated in subanalysis was a reduction in muscle strength for individuals with a poor appetite, compared with those with a good appetite.

Further research is needed to determine causality and its direction via longitudinal analysis of those with appetite change, associated alterations of gut microbiota composition and sarcopenia. These studies will require robust collection methods on confounders, which influence the gut microbiota including diet, medication, and frailty status. Furthermore, a more detailed analysis of the composition of the microbiota, including the *Lachnospira* genus as identified in this analysis, in relation to sarcopenia diagnosis and treatment responsiveness. This would inform intervention strategies with the potential to target the gut microbiota in order to manage anorexia of ageing and prevent onset or progression of sarcopenia.

## Author contributions

N.J.C., R.B., M.N.L., H.C.R. and C.S. performed the conceptualization and methodology; R.B. and P.M.W obtained the software used; R.B. conducted the final analysis; N.J.C., M.N.L., P.M.W., and R.B. carried out the data Curation; N.J.C. and R.B. were responsible for the writing—original draft preparation; N.J.C., R.B., M.N.L., H.C.R., and C.S. did the writing—review & editing; C.S. and H.C.R. made the supervision in this study.

## Funding

N.J.C and H.C.R receive support from the National Institute of Health Research (NIHR) Southampton Biomedical Research Centre. H.C.R receives support from the NIHR Applied Research Collaboration (ARC) Wessex. M.N.L is funded by an NIHR Doctoral Fellowship (RE160685). TwinsUK is funded by the Wellcome Trust (WT081878MA), Medical Research Council, European Union, the NIHR‐funded BioResource, Clinical Research Facility and Biomedical Research Centre based at Guy's and St Thomas' NHS Foundation Trust in partnership with King's College London. This work was also supported by the Chronic Disease Research Foundation. The views expressed are those of the authors and not necessarily those of the NHS, the NIHR or the Department of Health and Social Care.

## Conflict of interest

Natalie Cox, Ruth Bowyer, Mary Ni Lochlainn, Philippa Wells, Helen Roberts, and Claire Steves declare that they have no conflict of interest.

## Supporting information


**Figure S1.** Distributions and case‐control paired differences of covariates used to match cases to nearest controls (euclidean distance) for: A‐B SNAQ, C‐D Age at microbiota sample, E‐F Body Mass Index (BMI), G‐H Dietary quality as captured by the Healthy Eating Index (HEI), I‐J Estimated kilocalorie intake, K‐L Frailty Index (fi) M‐N Index of Multiple Deprivation (IMD), O‐P Difference in time between microbiome sample and SNAQ, Q‐R Sequencing depth/library size of 16s samples.

**Figure S2.** Weighted UniFrac distances within cases, controls, and paired differences between case and control (Paired‐CC).

**Table S1.** Supporting Information.
