# Supplementary figures and images for: The composition of the gut microbiome differs among community dwelling older people with good and poor appetite
Source: J Cachexia Sarcopenia Muscle. 2021 Feb 13;12(2):368–77. doi: 10.1002/jcsm.12683 (PMC8061352; doi:10.1002/jcsm.12683)

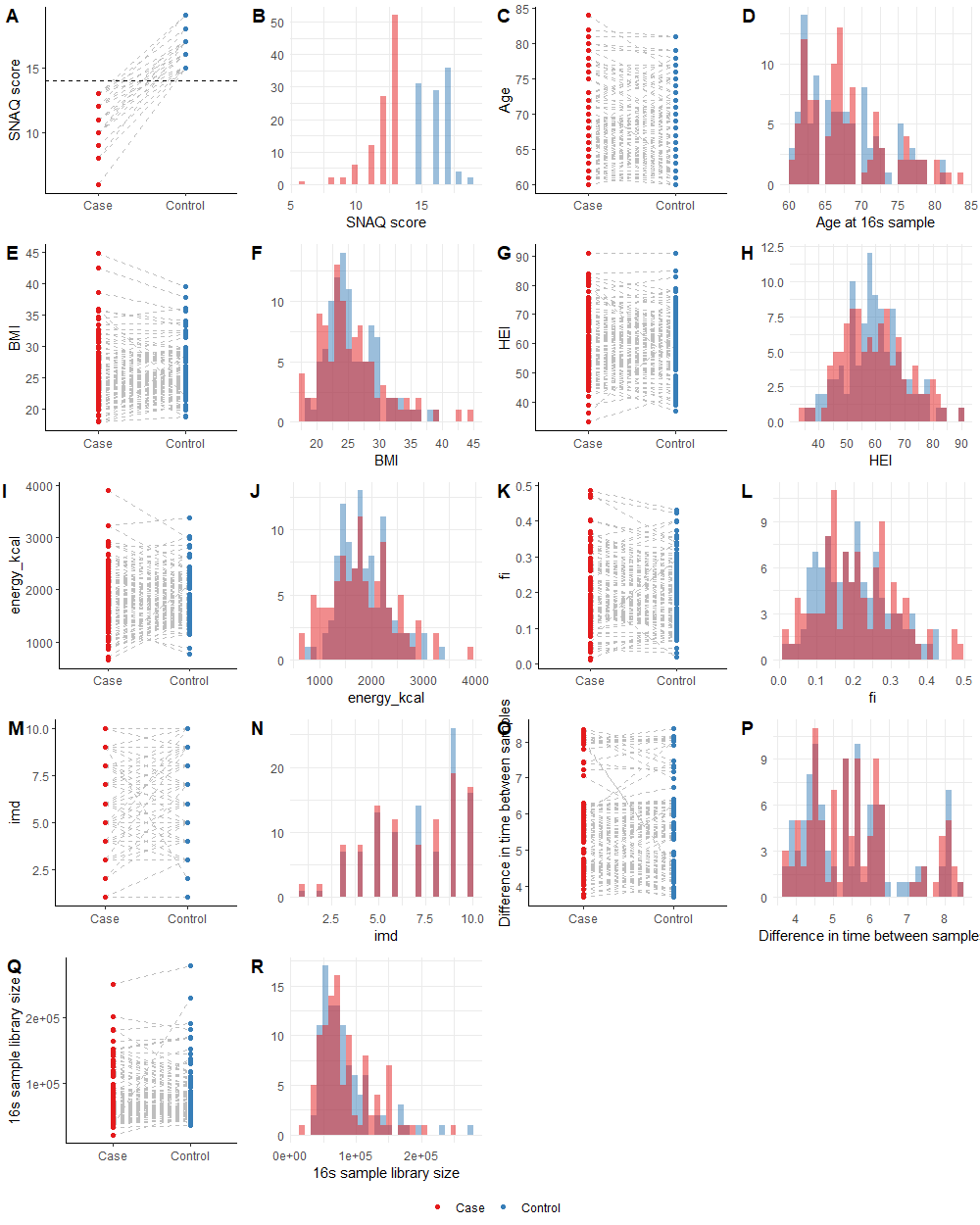

Supplement: Supplementary file 1 — Figure S1. Distributions and case‐control paired differences of covariates used to match cases to nearest controls (euclidean distance) for: A‐B SNAQ, C‐D Age at microbiota sample, E‐F Body Mass Index (BMI), G‐H Dietary quality as captured by the Healthy Eating Index (HEI), I‐J Estimated kilocalorie intake, K‐L Frailty Index (fi) M‐N Index of Multiple Deprivation (IMD), O‐P Difference in time between microbiome sample and SNAQ, Q‐R Sequencing depth/library size of 16s samples. [file JCSM-12-368-s002.tiff]

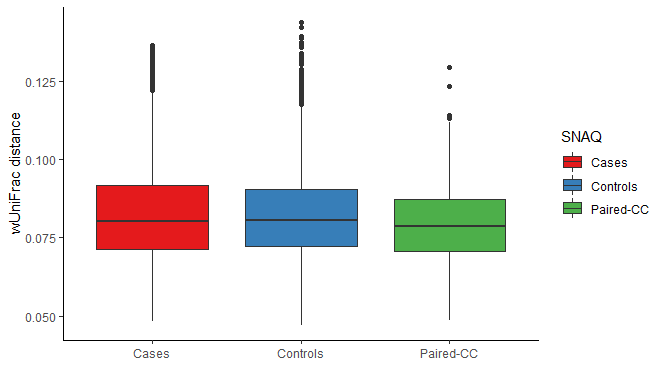

Supplement: Supplementary file 2 — Figure S2. Weighted UniFrac distances within cases, controls, and paired differences between case and control (Paired‐CC). [file JCSM-12-368-s001.tiff]
